# Work absences among hospital cleaning staff during the SARS-CoV-2 (COVID-19) pandemic

**DOI:** 10.47626/1679-4435-2020-871

**Published:** 2022-03-30

**Authors:** João Luiz Grandi, Cristiane de Oliveira Silva, Dulce Aparecida Barbosa

**Affiliations:** 1 Gerência de Risco e Meio Ambiente, Hospital São Paulo, Universidade Federal de São Paulo (Unifesp), São Paulo, SP, Brazil.; 2 Disciplina de Enfermagem Clínica e Cirúrgica, Unifesp, São Paulo, SP, Brazil.

**Keywords:** SARS-CoV-2, COVID-19, pandemics, occupational health, housekeeping, hospital

## Abstract

**Introduction:**

Absenteeism justified by sick leaves are valuable indicators of workers’ health conditions.

**Objectives:**

To analyze hospital cleaning staff sick leaves during the COVID-19 pandemic.

**Methods:**

This retrospective cohort study included employees who presented a medical sick leave certificate justifying at least 1 missed day of work during the first wave of the COVID-19 pandemic. Data were collected from March 24 to December 31, 2020 at a teaching hospital.

**Results:**

A total of 199 workers who presented 689 medical certificates were included in the sample. The sample was 88.4% women. The mean ages for suspected COVID-19 cases and all other cases were 39.7 years and 40.9 years, respectively. Suspected COVID-19 cases involved longer leaves (mean 5.82 [SD, 3.35] days missed) and more medical sick leave certificates (mean 4.25 [SD, 3.13] certificates per worker) than other causes. Among suspected cases, 32.1% worked in critical areas of the hospital. Of the 83 RT-PCR tests performed, 24.1% were positive, with 80% of these employees working in semi-critical or administrative areas; 15% of workers who tested positive developed the severe form of the disease.

**Conclusions:**

Among workers who underwent RT-PCR testing, the rate of positive results was low. Most positive cases occurred in younger women who worked in non-critical units (ie, units involving no direct patient contact or without aerosol-generating procedures). The mean number of missed days was higher among suspected COVID-19 cases (7.85 days [SD, 4.05]). The use of individual protective equipment was common among these employees, and they were continuously trained.

## Introduction

The emergence of SARS-CoV-2, a new human coronavirus capable of taking on pandemic proportions in just a few months, was first reported at the end of 2019 in Wuhan, China. Since then, it has become the most discussed global health issue in all media^1^ due to the speed and magnitude of its dissemination.

SARS-CoV-2 has greatly affected the world economy, leading the governments and health authorities of various countries in Europe and the Americas, including Brazil, to implement social isolation policies, initially in large cities affected by the pandemic. Although social isolation is one health measure used to contain the rapid spread of the virus, other measures have also been necessary, such as opening only essential services and maintaining the health care network, which basically consists of front-line health professionals.^2^

Thus, both professional activities and the work environment are potential exposure factors for the virus. In Singapore, 68% of initial cases of community contamination were associated with professional practice.^3^ A Greek study estimated that 10% of diagnosed COVID-19 cases occurred in health professionals.^4^

Around the world, thousands of health professionals took sick leaves and many died as a result of COVID-19 infection. In March 2020, in China, the epicenter of the pandemic, it was estimated that approximately 3,300 health professionals were infected, of whom 22 (0.6%) died.^5^ A retrospective study of clinical doctors and nurses in Wuhan found that 72 were infected with COVID-19.^6^ In Italy, the first Western country hit by the pandemic, Chirico et al.^7^ estimated that more health professionals tested positive for SARS-CoV-2 than in China; As of April 2020, of the approximately 12,000 health professionals who tested positive for COVID-19, 80 doctors and 25 nurses died.

However, other professional activities can play a relevant role in the containment or spread of the virus, and understanding how such activities are processed is essential for preventing illness.^8^ Among front-line health professionals, hospital cleaning staff stand out since their function is to disinfect hospital environments, especially places where COVID-19 patients are treated. They follow meticulous hygiene protocols, collecting waste and disinfecting surfaces to create barriers to the spread of the virus in the environment.^9^

Thus, due to the lack of specific literature on the importance of hospital cleaning staff for containing cross-infection and the spread of SARS-CoV-2 in the hospital environment, the present study was developed to analyze the reasons for work absenteeism among hospital cleaning staff during the SARS-CoV-2 (COVID-19) pandemic.

## Methods

### Study design, period, and location

This retrospective cohort study was conducted from March 24 to December 31, 2020, at the Hospital São Paulo, a high complexity, teaching and research hospital of Universidade Federal de São Paulo (Unifesp). This hospital is a reference center for COVID-19 treatment. In 2020, during the pandemic, the hospital maintained 642 beds, of which 89 (44 intensive care and 45 general ward) were reserved exclusively for COVID-19 patients.

### Population/sample

All employees formally hired (ie, according to the Consolidação das Leis Trabalhistas) to perform cleaning and disinfection work were included in the study. All absences of at least 1 full day due to medical or dental conditions were assessed according to the reasons described in the medical sick leave certificates.

### Inclusion criteria

All hospital cleaning staff who presented at least one medical sick leave certificate during the study period were included.

### Exclusion criteria

Employees on maternity or paternity leave during the study period were excluded. Leaves due to work accidents or causes unrelated to health conditions were also not included in this study.

### Study protocol

All medical certificates received when employees returned to work after a sick leave were scanned and sent to the Occupational Safety and Medicine Service. The date the leave began, the extent of the leave, and the clinical indication for the leave were extracted, as were the employee’s sex, age, shift, and work unit. Based on the clinical diagnosis in the medical certificates, the medical data were analyzed according to the International Statistical Classification of Diseases and Related Health Problems - 10th Revision (ICD-10).^10^ For certificates without proper ICD categorization, the reason for the leave was classified as “contact with health services”.

### Statistical analysis

For the descriptive analysis of continuous variables, measures of central tendency (mean and median) and variability (minimum, maximum, and standard deviation) were used. For categorical variables regarding suspected COVID-19 cases and serology results, Pearson’s chi-square test was used and, when necessary, Fischer’s exact test or the likelihood ratio test. For continuous variables regarding suspected COVID-19 cases and test results, the Mann-Whitney or McNemar test was used, in addition to calculating the kappa coefficient. In all cases, the significance level was set at 5% (p < 0.05). The data were exported from Microsoft Excel to SPSS^®^ version 22 for analysis.

### Ethical aspects

This study was registered in Plataforma Brasil, was approved by Unifesp Research Ethics Committee (number 4,274,757), and met the requirements of National Health Council Resolutions 466/2012 and 510/2016.

## Results

The sample consisted of 199 hospital cleaning staff, who presented 689 medical sick leave certificates.

Of the hospital cleaning staff who took a sick leave, 176 (88.4%) were female and 23 (11.6%) were male. Of those who took at least 1 sick leave due to suspected COVID-19 infection, 100 (56.8%) were women and 11 (47.8%) were men. There were no significant age differences between the groups (p = 0.8417), but 37.7% (75) of suspected COVID-19 cases were between 30 and 39 years of age. Among employees under 30 years of age, 60.7% (17) of the medical certificates reported an ICD classification related to SARS and 39.3% (11) were related to other causes. Among those aged 50 years or older, there was no significant difference between suspected COVID-19 cases and other causes. The mean age was 38.6 for suspected COVID-19 cases and 41.5 years all other causes, which suggests that younger employees took more sick leaves due to suspected COVID-19 infection. The mean number of missed days was higher for suspected COVID-19 cases than other causes: 4.25 (standard deviation [SD], 3.13) vs 2.47 (SD, 1.81), respectively, which was a significant difference (p ≤ 0.0001). Of suspected/confirmed COVID-19 cases, the mean number of missed days was 5.82 (SD, 3.35) vs 3.82 (SD, 4.95) for other causes (p ≤ 0.0001) (Table 1).

**Table 1:** Characteristics of employees who took sick leaves between March and December 2020 according to COVID-19 status, São Paulo, Brazil, 2021 (n = 199)

Variables	Suspected COVID-19 case				Total		p-value
	No		Yes				
	n	%	n	%	n	%	
Sex							
Female	76	43.2	100	56.8	176	88.45	0.4142*
Male	12	52.2	11	47.8	23	11.55	
Total	88	44.2	111	55.8	199	100.00	
Age range (years)							
< 30	11	39.3	17	60.7	28	10.05	
30 to 39	32	42.7	43	57.3	75	37.69	0.8417*
40 to 49	28	45.2	34	54.8	62	31.16	
50 and +	17	50.0	17	50.0	34	17.09	
Total	88	44.2	111	55.8	199	100.00	
Age							
Mean (SD)	40.97 (9.87)		39.7 (9.9)		40.26 (9.98)		0.3843†
Median	41.5		38.6		39.2		
Min-max	22.6-64.3		20.1-61.7		20.1-64.3		
Total	88		111		199		
Number of medical certificates							
Mean (SD)	2.47 (1.81)		4.25 (3.13)		3.46 (2.77)		< 0.0001†
Median	2		5		4		
Min-max	1-12		1-15		1-15		
Total	88		111		199		
Days missed							
Mean (SD)	3.82 (4.95)		5.82 (3.35)		4.96 (4.22)		< 0.0001†
Median	2.7		5		4		
Min-max	1-40.3		1.7-21.6		1-40.3		
Total	88		111		199		

SD = standard deviation.

* Chi-square test.

† Mann-Whitney test.

A total of 689 medical certificates were analyzed, of which 308 (44.7%) were for workers in semi-critical sectors of the hospital, and 71 (23.1%) were for suspected COVID-19 cases. Regarding seasonality, most of the sick leaves (249) occurred during the initial period of the pandemic, March 24 to June 21, 2020 (ie, autumn in Brazil), and there was no significant difference between occurrences in the winter and spring (163 and 171, respectively). However, regarding suspected COVID-19 cases, 99 (39.8%) occurred in the autumn and 127 (80.1%) in the spring (p ≤ 0.0001).

Regarding ICD-10 classification, 253 medical certificates reported diseases of the respiratory system. COVID-19 was suspected in 177 (70%) of these, while the other 76 (30%) involved respiratory diseases unassociated with SARS. The mean sick leave was 7.85 days (min-max 1-20 days) for those with suspected or confirmed COVID-19 infection and 3.48 days. (min-max 1-7 days) for all other causes (Table 2).

**Table 2:** Distribution of labor variables, work sector, length of sick leave, and leave due to suspected COVID-19 or not among all medical certificates received from March to December 2020, São Paulo, 2021 (n = 689)

Variables	Suspected COVID-19 case				Total		p-value
	No		Yes				
	n	%	n	%	n	%	
Work sector							
Critical (aerosols)	131	67.9	62	32.1	193	100.0	0.0544*
Semicritical	237	76.9	71	23.1	308	100.0	
Non-critical	144	76.6	44	23.4	188	100.0	
Total	512	74.3	179	25.7	689	100.0	
Season medical certificate emitted							
Fall	150	60.2	99	39.8	249	100.0	
Winter	129	79.8	33	20.4	162	100.0	
Spring	127	80.1	34	19.9	171	100.0	< 0.0001*
Summer	96	89.7	11	10.3	107	100.0	
Total	512	74.3	177	25.7	689	100.0	
ICD-10 classification							
Respiratory system	76	30.0	177	70.0	253	100.0	< 0.0001^†^
Other	436	100.0	-	-	436	100.0	
Total	512	74.31	177	25.7	689	100.0	
Days missed							
Mean (SD)	3.48 (7.3)		7.85 (4.05)		4.61 (6.88)		< 0.0001^‡^
Median	2		7		3		
Min-max	1-14		1-20		1-20		
Total	508		177		689		

ICD-10 = International Statistical Classification of Diseases and Related Health Problems - 10th Revision; SD = standard deviation.

* Chi-square test.

† Likelihood ratio test.

‡ Mann-Whitney test.

Table 3 shows the results for nasopharyngeal swab samples submitted to reverse transcriptase polymerase chain reaction (RT-PCR) testing. Of the 83 tests performed, 20 (24.1%) were positive (16 [80%] women and 4 [20%] men). Positive results were less expressive among staff who worked in critical units (ie, involving direct care for patients with intubated airways or aerosol-generating procedures)(4.20%). Although the disease initially spread faster among older adults than the general population, in our sample only 4 (20%) positive cases occurred among staff aged at least 50 years.

**Table 3:** Distribution of labor variables, work sector, length of sick leave, and nasopharyngeal swab (RT-PCR) results for COVID-19 of hospital cleaning staff who were tested from March to December 2020, São Paulo, 2021 (n = 83)

Variables	Nasopharyngeal swab (RT-PCR)				Total		p-value
	Negative		Positive				
	n	%	n	%	n	%	
Sex							
Female	58	92.1	16	16.0	25	100.0	0.6512*
Male	5	7.9	4	33.3	24	100.0	
Total	63	77.5	20	24.1	83	100.0	
Work sector							
Critical (aerosols)	21	84.0	4	20.0	25	100.0	0.3639^†^
Semicritical	16	66.7	8	40.0	24	100.0	
Non-critical	26	76.6	8	40.0	34	100.0	
Total	63	75.9	20	24.1	83	100.0	
Age range (years)							
> 30	7	11.1	1	5.0	8	100.0	
30 to 39	26	41.2	8	40.0	30	100.0	0.8174^†^
40 to 49	19	30.1	7	35.0	22	100.0	
≥ 50	11	17.4	4	20.0	1	100.0	
Total	63	75.9	20	24.1	83	100.0	
ICD-10 classification							
Respiratory system	33	78.6	9	21.4	42	100.0	
Health service contact	15	91.8	1	6.3	16	100.0	
Musculoskeletal system	6	60.0	4	40.0	10	100.0	
Digestive system	4	57.1	3	42.8	7	100.0	
Nervous system	2	100.0	-	-	2	100.0	
Infectious diseases	1	100.0	-	-	1	100.0	
Mental disorders	1	100.0	-	-	1	100.0	
Skin and subcutaneous tissue	1	100.0	-	-	1	100.0	
Circulatory system	-	-	1	100.0	1	100.0	
Genitourinary	-	-	1	100.0	1	100.0	
Childbirth and the puerperium	-	-	1	100.0	1	100.0	
Total	63	75.9	20	24.1	83	100.0	
Age							
Mean (SD)	40.44 (9.84)		41.09 (8.98)		40.59 (9.59)		0.6497^†^
Median	39.1		40.9		39.1		
Min-max	21.9-62.4		21.8-56.3		21.8-62.4		
Total personnel	63		20		83		
Days missed							
Mean (SD)	4.64 (7.87)		5.21 (3.84)		4.78 (7.1)		< 0.0001^‡^
Median	3		5		3		
Min-max	1-60		1-14		1-60		
Total personnel	63		20		83		
Number of medical sick leave certificates							
Mean (SD)	3.51 (2.57)		3.44 (2.42)		3.49 (2.52)		0.9833^†^
Median	3		3		3		
Min-max	1-12		1-10		1-12		
Total personnel	63		20		83		

ICD-10 = International Statistical Classification of Diseases and Related Health Problems - 10th Revision ; SD = standard deviation; RT-PCR = reverse transcriptase polymerase chain reaction testing.

* Likelihood ratio test.

† Chi-square test.

‡ Mann-Whitney test.

Regarding positive COVID-19 results and the ICD-10 classifications reported in the medical certificates, 9 (21.4%) of the 42 respiratory system cases were positive, while 3(42.8%) of those classified as diseases of the digestive system were also positive.

Regarding the length of sick leave, the mean number of days was higher for COVID-19 patients: 5.21 vs 4.64 for non-COVID-19 patients, a significant difference (p ≤ 0.0001). Regarding the number of certificates per worker, there was no significant difference between those suspected COVID-19 patients and non-COVID-19 patients (p = 0.9833).

Table 4 shows the RT-PCR tests results for all 83 (79.2%) employees suspected of COVID-19 infection (ie, whose medical certificates indicated ICD-10 “diseases of the respiratory system” compatible with SARS). In 20 (24.1%) of these patients, the nasopharyngeal swab sample was reactive for COVID-19: 3 (15%) of these employees developed the severe form of the disease, 2 of whom were hospitalized in intensive care. There was no significant difference in the proportion of positives between suspected COVID-19 cases and the test results. The kappa coefficient indicated very poor agreement (< 0.2).

**Table 4:** Distribution of nasopharyngeal swab test results among hospital cleaning staff whose medical sick leave certificate reported diseases of the respiratory system (ICD-10) or suspected COVID-19, São Paulo, Brazil, 2021 (n = 83)

Variables	Nasopharyngeal swab						p-value	kappa*
	Non-reactive		Reactive		Total			
	n	%	n	%	n	%		
Suspected COVID-19								
Yes	23	27.7	8	9.6	31	37.3	0.0895^†^	0.029*
No	40	48.2	12	14.5	52	62.7		
Total	63	75.9	20	24.1	83	100.0		

ICD-10 = International Statistical Classification of Diseases and Related Health Problems - 10th Revision.

* Kappa coefficient.

† p-value = McNemar test.

## Discussion

Of the 287 permanent hospital cleaning staff included in this study, 199 (69.4%) presented at least one medical sick leave certificate during the quarantine in the city of São Paulo. In contrast, a study in Portugal reported that 453 (73.2%) of the 619 physical interviewed therapists interrupted their face-to-face activities in March 2020 due to the pandemic.^11^

Of the workers who took a sick leave in our study, 88.5% were female. This disproportion can be explained in light of Brazilian culture, in which hygiene and cleaning occupations are viewed as the province of women.^12^ Among workers who took sick leaves, 44.2% were suspected/confirmed cases of COVID-19 and 55.8% were unrelated to COVID-19. This corroborates Chinese studies by Zhang et al.^13^ and Lai et al.,^14^ in which more female than male health workers were suspected of COVID-19 infection.

Our sample was evenly distributed in age ranges between 30 and 50 years. However, at the extremes, approximately 10% (28) and 17% (34) were < 30 or ≥ 50 years, respectively. The mean ages of those on leave due to suspected COVID-19 vs all other causes were 38.6 and 41.5 years, respectively. A study of 30 doctors and nurses with COVID-19 at a university hospital in Jianghan, China found ages ranging from 21 to 59 years (mean 35 [SD, 8] years).^15^ Among 54 doctors hospitalized with COVID-19 in Wuhan, China, the mean age of those with the mild form was significantly higher than the severe form.^16^ However, 79.6% (43/54) of the cases were severe.

In a Brazilian study that analyzed 250,000 COVID-19 hospitalizations, the mean patient age was 60 years (SD, 17). However, in the northeast region, the authors found a higher proportion of patients aged at least 80 years.^17^ Among physicians hospitalized in Wuhan in 2020, the data suggest that the disease had a greater impact among the oldest and youngest members of the general population.^16^ Our sample confirms Chu et al. data^16^ in that we found 40% (8) aged between 30 and 39 years old and 35% (7) aged between 40 and 49 years.

Regarding COVID-19 cases according to hospital sector, of the 199 workers who presented medical sick leave certificates, 308 (44.7%) were from semi-critical areas, and, among suspected/confirmed COVID-19 cases, 62 (32.1%) were removed from critical areas. These data corroborate two other studies.^2,16^ In a study that analyzed 40 health professionals infected with SARS-CoV-2,^2^ 31 (77.5%) worked in clinical units and only 2 (5%) worked in intensive care units. Among 54 physicians hospitalized for COVID-19 in Wuhan, 39 (72.2%) worked in clinical units, 10 (18.5) worked in the medical technology department, and only 2 (3.7%) were physicians working in intensive care units.^16^

According to the ICD-10^10^ classifications described in our sample’s medical certificates, diseases of the respiratory system were the main reason (42 [50.6%] cases), followed by codes Z00-Z99 “Factors influencing health status and contact with health services”, then diseases of the musculoskeletal system (16 [19.2%]) and connective tissue disorders (10 [12.5%]). In a study conducted by our group prior to the pandemic, of 1,307 medical certificates analyzed, 282 (21.5%) were related to ICD codes Z00-Z99, and 271 (20.7%) were related to musculoskeletal or connective tissue disorders.^12^ Such data suggest that there may have been an inversion of the reasons for sick leave: prior to the pandemic, musculoskeletal disorders were recurrent, showing that this group of workers may be more prone to musculoskeletal diseases, such as work-related musculoskeletal disorders. During the pandemic, sick leaves due to respiratory disorders and suspected COVID-19 were more common. However, in another study of outsourced hospital cleaning staff in Curitiba, Paraná, Brazil, sick leaves were mostly short and due to codes Z290-298.^12^

Several authors have reported that caring for COVID-19 patients in the emergency room leads to anxiety and physical and mental exhaustion.^18,19^ Others have reported burnout, depression, and minor psychiatric disorders.^20^ A Vietnamese study conducted in 3 central hospitals in Hanoi at the beginning of the pandemic found depression rates ranging from 6.7 to 20.1% among health workers. The authors concluded that one factor for depression was working at the only local hospital that enacted a lockdown.^21^ However, in our study the number of sick leaves due to mental disorders (anxious and depressive conditions) was low, reported in only 17 (2.5%) of the medical certificates. During the Ebola epidemic, 39% of the health professionals in Sierra Leone reported anxiety.^22^ The authors this study pointed out that, in the context of pandemics, health workers must pay greater attention to their mental health^18,19^ and use strict prevention measures to reduce infection.

In the present study, 20 (24.1%) of the hospital cleaning staff who tested positive for COVID-19 in RT-PCR, 2 (10.0%) of whom developed the severe form of the disease, requiring intensive care unit hospitalization, and 1 (5%) other was hospitalized in a ward. Of these 3 cases, only one had comorbidities. The remaining 17 (85.0%) had the mild form of the disease and were treated at home. In the second quarter of 2020 in Madrid, of the 399 healthcare professionals tested with RT-PCR, 159 (39.9%) were positive for SARS-CoV-2.^23^

The severity of the disease may also vary according to the region and the period in which the studies were conducted. In Brazil, about 16% of those hospitalized in the northeast region had the severe form of the disease and required mechanical ventilation, compared to 8% in the southeast region under the same circumstances.^17^ In China, of the 54 doctors hospitalized at the Wuhan hospital, 11 (20.3%) had the mild form of COVID-19 and 40 (74%) had the severe form.^16^ In Greece, of 755 symptomatic health professionals, 454 (60%) were positive for COVID-19; of these, only 13 (2.87%) were hospitalized and there were no deaths.^4^

In 2 Chinese studies of health professionals conducted at the beginning of the pandemic, the authors reported that 2.3%^13^ were diagnosed with COVID-19 and that 3.1%^24^ were suspected or confirmed SARS-CoV-2 cases. Two other studies conducted months later reported infection rates of 3.5^25^ and 4.4%^26^ among health professionals. In a specialized nursing clinic in the United States, of 76 resident nurses, 48 (63.1%) tested positive for COVID-19 in RT-PCR.^27^

The particular vulnerability of the less fortunate to COVID-19 infection seems to be an example of social injustice and structural inequalities in the labor market, since many low-income individuals work in occupations considered essential and are thus exempt from social isolation (staying at home or remote work). In addition, many live in cramped or multifamily residences that do not allow for social distancing.

It is noteworthy that only 20% (4) of the COVID-19 cases in our sample worked in intensive care units, ie, where COVID-19 patients are treated. This might be explained by the exhaustive systematic training these professionals underwent. Many of these training sessions involved the entire health team in each sector, underscoring the importance of hygiene work and prevention among this group of workers. While significant progress has been made in implementing best practices for sanitizing and disinfecting high-risk COVID-19 environments, more concerted efforts are needed to further reduce the frequency of healthcare workers infected with COVID-19.

Special infection prevention and control measures are essential to prevent the spread of the disease in the workplace, and an emphasis should be given to staff training.^27^ However, in several countries, emergency room contamination is aggravated by a lack of personal protective equipment due to health system overloading and underfunding.^28^ However, personal protective equipment is essential for protective measures. In the present study, which included a homogeneous sample of workers, a further risk factor was that all of the workers were low income and had to use public transportation daily. The associated contamination factors suggest that, among these workers, SARS-CoV-2 may have been transmitted at both nosocomial and community levels.

### Study limitations and contributions

This study has some limitations, mainly due to the difficulty of obtaining confirmatory swab tests (RT-PCR) at the beginning of the pandemic. It is possible that, prior to the availability of the tests, some positive cases were not confirmed or corrected in the sample.

Data analysis was limited to medical sick leave certificates, which prevented determining a correlation between nosocomial and community contamination. Future studies should focus on rates of community infection among low-income workers who live in poor communities.

Our study helps clarify the disease’s behavior and dynamics in this group of workers, as well as its impact on labor. It also demonstrates that effective training measures can help reduce COVID-19 infection among frontline workers.

## Conclusions

This study provided insight into the dynamics of SARS-CoV-2 contamination among hospital cleaning staff. The sample, mostly female, had a mean age of 40.26 years; only 34 employees were considered to be in the risk group (≥ 50 years). We found variation in the mean number of medical certificates presented and length of sick leave, with both being higher among employees with suspected COVID-19. The sick leaves were mostly due to diseases of the respiratory system associated with flu syndrome. RT-PCR testing revealed lower positivity and longer sick leaves (mean 7.85 days) among suspected cases. We conclude that the infection risk among hospital cleaning staff can be mitigated by taking adequate precautions, including social isolation in suspected cases and providing adequate personal protective equipment, such as hats, aprons, NR-95 surgical masks, vinyl gloves, face shields, safety glasses, and long boots.
